# The prognostic value of LAYN in HPV-related head and neck squamous cell carcinoma and its influence on immune cell infiltration

**DOI:** 10.1007/s12672-024-00913-5

**Published:** 2024-03-02

**Authors:** Qingjuan Chen, Jiankang Chen, Zuzhuang Lu, Rui Nian, Wanjun Li, Zhongqiang Yao, Shangdong Mou, Ying Liu, Xia Cao, Wenjing He, Chenjing Zhu

**Affiliations:** 1 Department of Oncology, 3201 Hospital of Xi’an Jiaotong University Health Science Center, Hanzhong, 723000 Shaanxi China; 2 Department of Oncology, Yongchuan Hospital of Chongqing Medical University, Chongqing, 40016 China; 3 Department of Pathology, 3201 Hospital of Xi’an Jiaotong University Health Science Center, Hanzhong, 723000 Shaanxi China; 4 Department of Oncology, Xianyang Center Hospital, Xi’an, 712000 Shaanxi China; 5 Department of Radiation Oncology, Jiangsu Cancer Hospital & Jiangsu Institute of Cancer Research & Affiliated Cancer Hospital of Nanjing Medical University, 42 Baiziting, Nanjing, 210009 Jiangsu China

**Keywords:** LAYN, Prognosis, HPV, HNSCC, Immune cell infiltration

## Abstract

**Background:**

HPV-positive head and neck squamous cell carcinoma (HNSCC) exhibits different characteristics from HPV-negative tumors in terms of tumor development, clinical features, treatment response, and prognosis. Layilin (LAYN), which contains homology with C-type lectins, plays a critical role in tumorigenesis and cancer progression. However, the prognostic value of LAYN and the relationship between LAYN and immune infiltration levels in HPV-related HNSCC patients still require a comprehensive understanding. Herein, we aimed to assess the prognostic value of LAYN and to investigate its underlying immunological function in HPV-related HNSCC.

**Methods:**

Through various bioinformatics methods, we analyzed the data from The Cancer Genome Atlas (TCGA), Tumor Immune Estimation Resource (TIMER) and Gene Expression Profiling Interactive Analysis (GEPIA) databases to explore the potential underlying oncogenic impression of LAYN, including the relevance of LAYN to survival outcomes, clinicopathological factors, immune cell infiltration, and immune marker sets in HPV-related HNSCC. The expression levels of LAYN and HPV were also verified in HNSCC patient tissues.

**Results:**

LAYN was differentially expressed in a variety of tumors. The expression of LAYN in HNSCC was significantly higher than that in adjacent normal tissues (P < 0.0001), and high expression of LAYN was correlated with poor overall survival (OS) in HNSCC patients (Hazard Ratio (HR) = 1.3, P = 0.035). Moreover, LAYN expression level in HPV-positive HNSCC patients was significantly lower than that in HPV-negative patients, with HPV-positive HNSCC patients displaying a trend of favorable prognosis. In addition, the relationship between LAYN expression and immune infiltration levels in HPV-positive HNSCC group was less tightly correlated than that in HPV-negative HNSCC group, and there was a strong relationship between LAYN expression and markers of M2 macrophage (P < 0.001) and exhausted T cells (P < 0.05) in HPV-negative HNSCC. Kyoto encyclopedia of genes and genomes (KEGG) enrichment analysis suggested that LAYN potentially influenced tumor progression through HPV infection and other cancer-related pathways.

**Conclusions:**

LAYN might contribute to tumorigenesis via its positive correlation with immune checkpoint molecules and tumor-associated macrophages (TAMs). Our study might provide a novel prognostic biomarker and latent therapeutic target for the treatment of HPV-related HNSCC.

## Introduction

Head and neck squamous cell carcinoma (HNSCC) are a heterogeneous group of tumors derived from the mucosal epithelium in the oral cavity, pharynx and larynx [1, 2]. Main risk factors include exposure to cigarette smoke and/or alcohol, and infection with high-risk types of human papillomavirus (HPV), primarily HPV-16 [3]. Thus, HNSCC can be classified as HPV-negative or HPV-positive, with different mutation profiles, molecular characteristics, immune landscapes, and clinical prognoses [4, 5]. Studies have shown that the infiltration of immune cells, especially T cells, was more abundant, and the mutational load of neo-antigens was significantly lower in HPV-positive HNSCC patients [6]. Therefore, HPV-positive HNSCC could act as "hot tumors" and were sensitive to radiotherapy, chemotherapy, and immunotherapy [7, 8].

Layilin (LAYN), first reported in 1998, was a 55 kDa transmembrane protein that located on chromosome 11 and shared homology with type C lectin [9]. It was known to be one of the receptors for hyaluronic acid (HA) which was a kind of glycosaminoglycan synthesized by tumor cells and accumulated in tumor matrix widely [10]. Through binding with HA oligosaccharides, LAYN participated in cell adhesion, movement and migration [10–12]. Previous studies have shown that LAYN regulated the immune balance among tumor-associated macrophages (TAMs), dendritic cells, and regulatory T cells (Tregs) in colon and gastric cancer tissues, and affected tumor cell progression [13, 14]. In addition, LAYN was identified as a Treg cell signature gene, and its high expression was correlated with poor prognosis in patients with non-small-cell lung cancer and colorectal cancer [14, 15].

There is a lack of relevant research on the prognostic value of LAYN in HNSCC patients and the relationship between LAYN and immune infiltration in HPV-related HNSCC. In the present study, we first visualized the expression landscape of LAYN in pan-cancer. The correlations between LAYN and clinicopathological factors, especially HPV infection status, and the relationship between LAYN and immune infiltration levels and immune marker sets was analyzed. Furthermore, the expression and prognosis patterns of HPV and LAYN in a total of 61 patients who underwent curative surgery for HNSCC were explored. Our findings might provide novel insights into the role of LAYN in HPV-related HNSCC, and expand the understanding of the underlying mechanism between LAYN and tumor-immune interactions.

## Methods and materials

### Clinical characteristics

A total of 61 patients who underwent curative surgery for HNSCC at 3201 Hospital (Hanzhong, China), from January 2012 to January 2021, were enrolled in this study. There were 44 cases of oral cancer, 11 cases of oropharyngeal cancer, and 6 cases of other types of cancer. Formalin-fixed paraffin-embedded tumor specimens were obtained. The clinical characteristics of the patients, including their age, gender, histopathological grade, TNM stage and metastasis location were collected. All patients included in this study were informed about the use of residual human corporal materials for clinical research, and written informed consents were obtained. This study was approved by the Institutional Review Board of 3201 Hospital (identification code: [2023]010), and carried out according to the provisions of the Helsinki Declaration.

### Tumor immune estimation resource (TIMER) database analysis

TIMER database is a comprehensive resource that utilizes RNA sequencing data from TCGA to evaluate the abundance of immune infiltration in various types of tumors. The database can be accessed at https://cistrome.shinyapps.io/timer/ and its analysis process was described in detail by Liu [16, 17]. The Diff Exp, Gene, Correlation, and survival modules were employed. The correlation of LAYN expression and the abundance of six types of immune infiltrating cells, including B cells, CD8 + T cells, CD4 + T cells, macrophages, neutrophils, and dendritic cells, were analyzed.

### Gene correlation analysis in gene expression profiling interactive analysis (GEPIA)

GEPIA (http://gepia.cancer-pku.cn/) is an interactive online platform that allows for gene expression analysis. Using tumor sample information from TCGA and normal sample information from the TCGA and Genotype-Twassue Expression (GTEx) Projects, the correlation between LAYN expression and multiple immune cell markers was identified [18]. Immune gene markers were selected from the Cell Marker database (http://biocc.hrbmu.edu.cn/CellMarker/). These gene markers include markers of B cells (CD19 and CD79A), CD8 + T cells (CD8A and CD8B), T cells (general) (CD3D, CD3E, and CD2), T-helper 1 (Th1) cells (T-bet, STAT4, STAT1, IFN-γ, and TNF-α), T-helper 2 (Th2) cells (GATA3, STAT6, STAT5A, and IL13), follicular helper T cells (Tfh) (BCL6 and IL21), T-helper 17 (Th17) cells (STAT3 and IL17A), Tregs (FOXP3, CCR8, STAT5B, and TGFβ), exhausted T cells (PD-1, CTLA4, LAG3, TIM-3, and GZMB), Monocytes (CD115 and CD86), tumor-associated macrophages (TAM) (CCL2, CD68 and IL10), M1 macrophages (INOS, IRF5, and COX2), M2 macrophages (CD163, MS4A4A, and VSIG4), neutrophils (CD66b, CD11b, and CCR7), natural killer (NK) cells (KIR2DL1, KIR2DL3, KIR2DL4, KIR3DL1, KIR3DL2, KIR3DL3, and KIR2DS4), and dendritic cells (HLA-DPB1, HLA-DQB1, HLA-DRA, HLA-DPA1, BDCA-1, BDCA-4, and CD11c).

### Immunohistochemistry of HPV and LAYN

To detect LAYN and HPV expression in a total of 61 HNSCC patients, SP immunohistochemistry was conducted in accordance with the manufacturer's instructions. LAYN primary antibody was commercially available from HUABIO, China, and the antibody dilution was set to 1/200. HPV primary antibody was ready-to-use and commercially available from Maixin BIOTECH, Fuzhou, China. The deparaffinized and rehydrated slides were incubated with the primary antibodies for 60 min, followed by thorough washing with the secondary antibody for 15 min, streptavidin for 10 min, and liquid DAB for 5 min. Cases were considered positive if a dark brown reaction was observed in the tumor cells.

### Pathway enrichment analysis

Gene Ontology (GO) and Kyoto Encyclopedia of Genes and Genomes (KEGG) analysis were conducted as previously described [19]. R software version 4.0.5 (https:// www.r-project.org/) was used for visualization.

### Statistical analysis

Student’s T-tests and Chi-square tests were utilized to determine the differences between two groups. Kaplan–Meier survival analysis was employed to assess survival. The correlation of gene expression was measure by Spearman's correlation. Graphs and charts were created using GraphPad Prism. A P < 0.05 was considered statistically significant.

## Results

### Differential mRNA expression level of LAYN in pan-cancer and its relationship with the prognosis of HNSCC

**Fig. 1:**
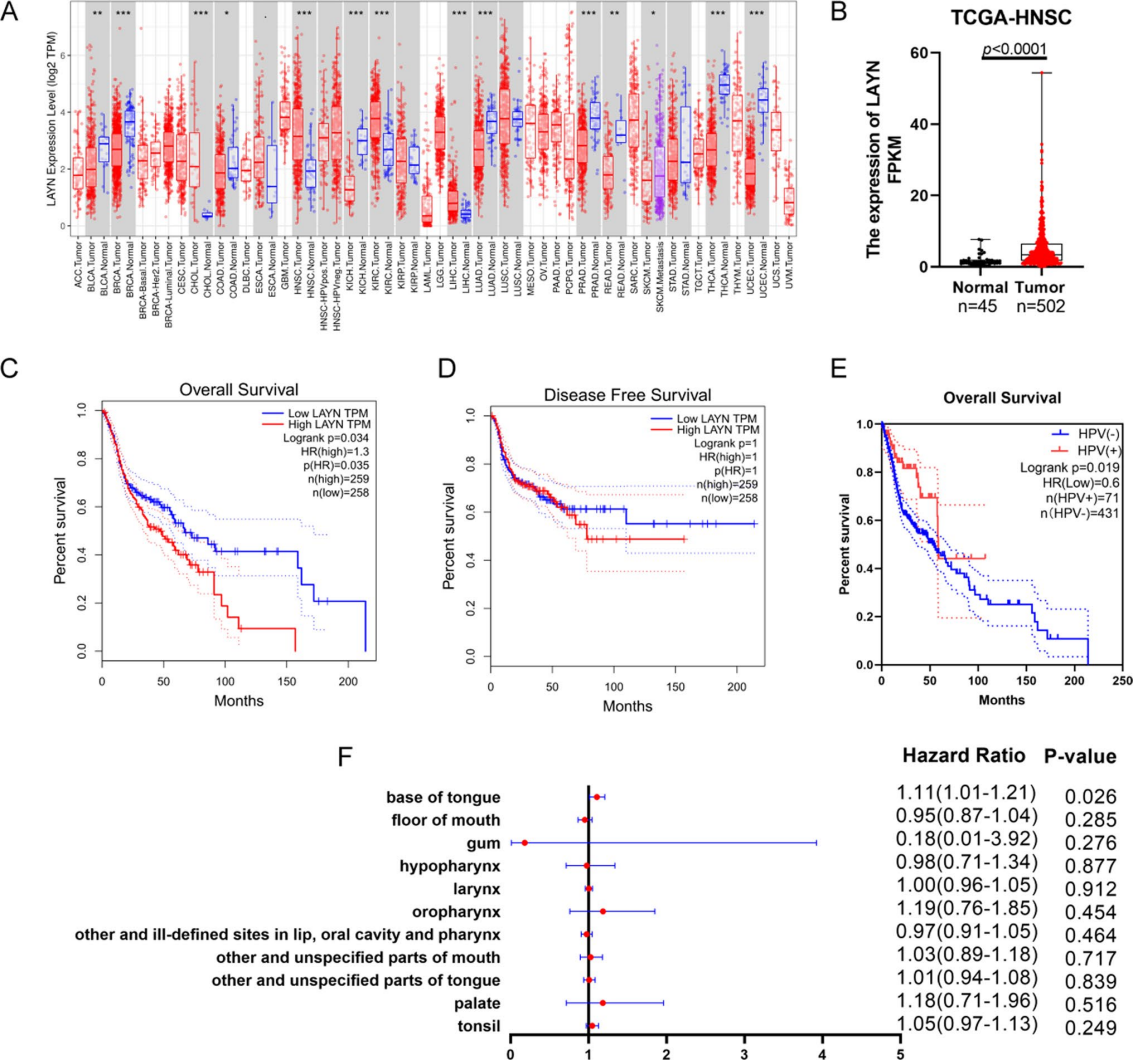
The differential expression of LAYN in diverse tumors and its role in the prognosis of HNSCC. **A** LAYN expression in different tumor types from the TCGA database was explored with TIMER (*P < 0.05, **P < 0.01, ***P < 0.001). **B** LAYN expression in HNSCC was explored in TCGA database. **C** OS survival curves of HNSCC patients with high and low LAYN expression. **D** DFS survival curves of HNSCC patients with high and low LAYN expression. **E** OS survival curves of HPV-positive and HPV-negative HNSCC patients. **F** Forest plot of LAYN expression and survival in common HNSCC subtypes. *OS* overall survival; *DFS* disease-free survival

We employed TIMER database to investigate the variances of LAYN mRNA expression levels between tumor and normal tissues in a wide range of cancer types. The analysis indicated that LAYN expression was higher in HNSCC, hepatocellular carcinoma, cholangiocarcinoma, and renal clear cell carcinoma than in their respective adjacent normal tissues. In contrast, lower expression of LAYN was observed in bladder urothelial cancer, breast cancer, colorectal cancer, kidney chromophobe, lung adenocarcinoma, prostate cancer, thyroid cancer, and endometrial cancer compared with adjacent normal tissues (Fig. 1A). The increased expression of LAYN in HNSCC was also confirmed in TCGA (P < 0.0001, Fig. 1B). These results implied that the expression of LAYN in pan-cancer was heterogeneous.

We further examined whether LAYN expression was correlated with the prognosis of HNSCC patients. Survival analyses revealed that high LAYN expression worsened the overall survival (OS) of HNSCC patients (Fig. 1C) (HR = 1.3, P < 0.05), while no difference existed in disease-free survival (DFS) with two curves intersecting between the LAYN-high and LAYN-low groups (HR = 1, P = 1) (Fig. 1D). Additionally, we explored whether HPV infection status was associated with patients’ survival, and found that the survival of HPV-positive patients was significantly better than that of HPV-negative patients (HR = 0.6, P < 0.05) (Fig. 1E). Through forest plot, we found that among common head and neck tumors, the expression of LAYN was only correlated with the survival of the base of tongue cancer patients with statistical difference (HR = 1.11, 95% CI 1.01–1.21, P < 0.05) (Fig. 1F). Therefore, LAYN expression could be an independent prognostic factor that led to the poor prognosis of HNSCC, especially the base of tongue cancer.

### The relationship between LAYN expression and clinicopathological factors in HNSCC

**Fig. 2:**
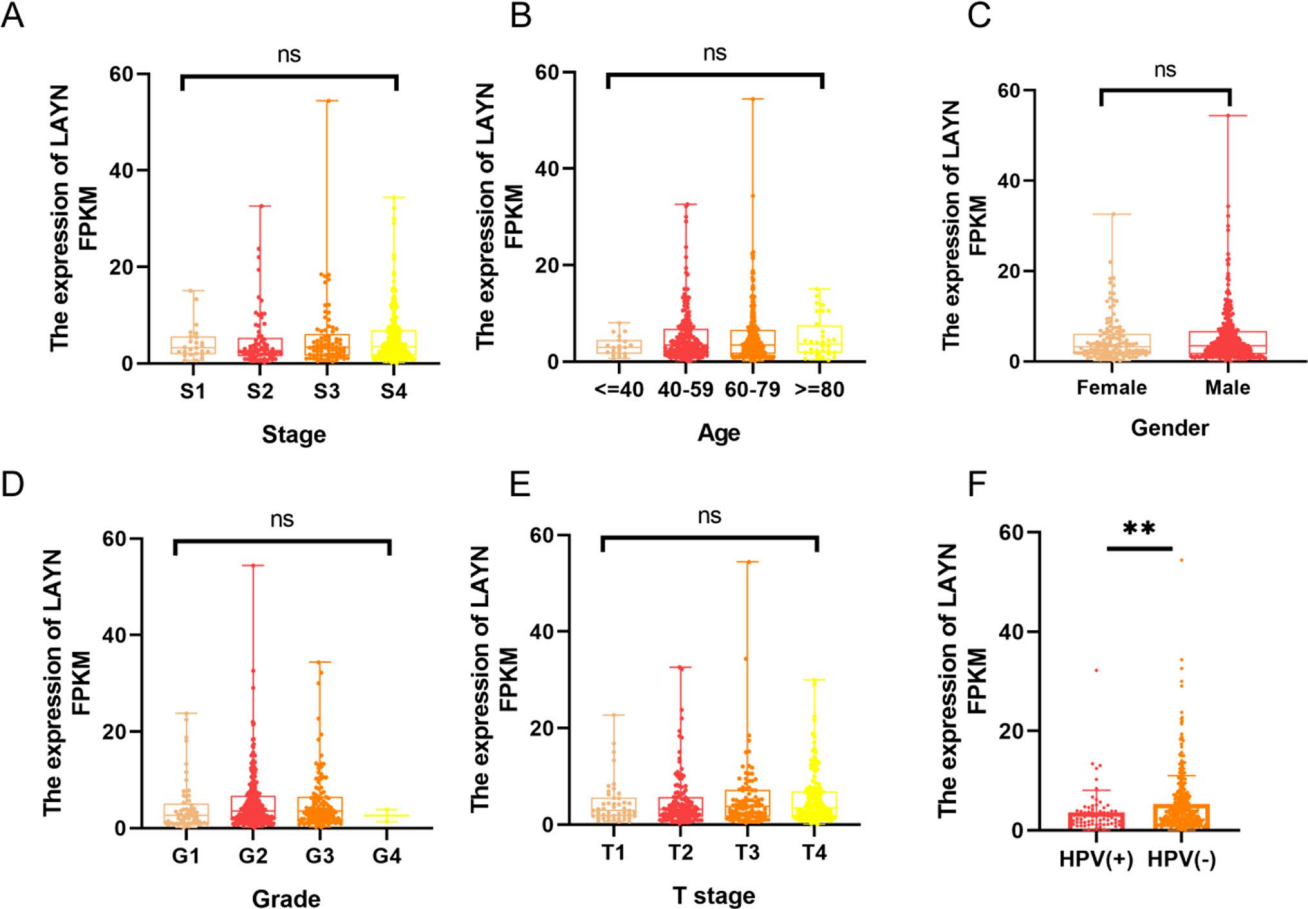
**A**–**F** The relationship between LAYN expression and clinicopathological factors in HNSCC patients. *ns* not significant, *P < 0.05, **P < 0.01, ***P < 0.001

To better understand the relevance and underlying mechanisms of LAYN expression in HNSCC patients, we analyzed the relationship between LAYN expression and clinical characteristics of HNSCC. Using R software to analyze data from TCGA database, we found that no correlation existed between LAYN expression and age, gender, clinical stage, T stage, and tumor grade, respectively (Fig. 2A–E). However, LAYN expression was associated with HPV infection status with statistical significance (P < 0.05), with LAYN expression in HPV-negative patients higher than that in HPV-positive patients (P < 0.01) (Fig. 2F).

### LAYN was significantly related to immune infiltration in HNSCC

**Fig. 3:**
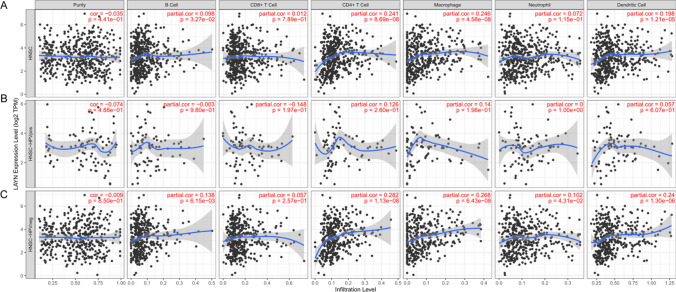
The correlation of LAYN expression with immune infiltration levels in **A** HNSCC, **B** HPV-positive HNSCC and **C** HPV-negative HNSCC patients

To explore the potential mechanisms by which LAYN influenced prognosis, correlation analyses were conducted between LAYN expression and tumor immunity based on tumor purity, immune cells, and immune checkpoint molecules using TIMER database. As was shown in Fig. 3A, after adjusting for the influence of tumor purity, LAYN expression was positively associated with the infiltrating levels of B cells (P < 0.05), CD4 + T cells (P < 0.001), macrophages (P < 0.001), and dendritic cells (P < 0.001) in HNSCC tumor tissues, while the correlation was not statistically significant with CD8 + T (P = 0.79) and neutrophils (P = 0.12).

We further analyzed the correlation between LAYN expression and immune cells in HPV-positive and HPV-negative HNSCC patients. Overall, the relationship between LAYN expression and immune markers in HPV-positive HNSCC group was less tightly correlated than that in HPV-negative HNSCC group (Table 1). In HPV-positive HNSCC patients, no correlation between LAYN expression and various immune cells was observed (P > 0.05) (Fig. 3B). However, in HPV-negative HNSCC patients, LAYN was positively correlated with infiltrating B cells (P < 0.01), CD4 + T cells (P < 0.001), macrophages (P < 0.001), neutrophils (P < 0.05), and dendritic cells (P < 0.001) in tumor tissues. The correlation between LAYN and CD8 + T cells in HPV-negative HNSCC patients was not statistically significant (P = 0.26) (Fig. 3C) (Table 1). These findings strongly suggested that LAYN played a specific role in immune cell infiltration in HPV-negative HNSCC patients.

**Table 1:** Correlation analysis between LAYN and immune cells in HPV-related HNSCC through TIMER database

Gene markers	HNSCC-HPVpos		HNSCC-HPVneg	
	r	P	r	P
Purity	− 0.074061577	0.487844009	− 0.009461608	0.850185829
B Cell	− 0.002919682	0.979626543	0.137813783	0.006145428
CD8 + T Cell	− 0.147732289	0.196784596	0.05734813	0.2567129
CD4 + T Cell	0.125730622	0.260362945	0.282344881	1.12774E−08
Macrophage	0.140207704	0.197893249	0.268220214	6.42732E−08
Neutrophil	2.8203E−06	0.999979561	0.102370792	0.043064051
Dendritic Cell	0.057318251	0.606763953	0.240408127	1.29771E−06

Purity: correlation adjusted for tumor purity; r: value of Spearman’s correlation

### Correlation analysis between LAYN expression and immune marker sets

In order to further explore the role of LAYN in the tumor immune process and its potential as a target for immunotherapy, we analyzed the correlation between LAYN expression and various immune marker sets of immune cells in HPV-related HNSCC through GEPIA database. All the markers were used to characterize immune cells, including markers of B cells, CD8 + T cells, CD4 + T cells, monocytes, macrophages, neutrophils, NK cells, dendritic cells and different functional T cells (Th1, Th2, Tfh, Th17, Treg, and exhausted T cells).

We found that the expression level of LAYN was associated with 30 out of 57 immune cell markers in HNSCC, 2 out of 57 in HPV-positive HNSCC, and 31 out of 57 in HPV-negative HNSCC with statistical significance (Table 2). The elevated LAYN expression level was associated with multiple immune cell markers in HNSCC, especially HPV-negative HNSCC, including markers of monocytes, TAMs, M1 macrophages, M2 macrophages, neutrophils, dendritic cells, and exhausted T cells (P < 0.05). However, In HPV-positive HNSCC patients, nearly no correlation between LAYN expression and various immune cell markers was observed, which was in consistent with our aforementioned results. Specifically, CCR8 of Treg cells, revealed strong correlation with LAYN in HNSCC, HPV-positive HNSCC and HPV-negative HNSCC patients (P < 0.01). These results indicated that LAYN might act as an immunomodulatory gene that involved in a variety of biological functions, including immune cell activation, antigen presentation, macrophage polarization and regulation of the T cells in HPV-related HNSCC.

**Table 2:** Correlation analysis between LAYN expression and immune marker sets of various immune cells in HPV-related HNSCC through GEPIA database

Description	Gene markers	HNSCC(n = 520)		HNSCC-HPVpos(n = 97)		HNSCC-HPVneg(n = 419)	
		r	P	r	P	r	P
CD8 + T cell	CD8A	− 0.008	0.859	− 0.075	0.461	0.030	0.540
	CD8B	− 0.017	0.703	− 0.103	0.311	0.032	0.508
T cell (general)	CD3D	0.033	0.448	− 0.029	0.776	0.070	0.151
	CD3E	0.085	0.052	0.033	0.750	0.122	0.012
	CD2	0.069	0.117	0.044	0.663	0.097	0.048
B cell	CD19	0.036	0.414	− 0.040	0.695	0.074	0.129
	CD79A	0.095	0.030	0.075	0.462	0.126	0.010
Monocyte	CD86	0.231	***	0.136	0.180	0.257	***
	CD115 (CSF1R)	0.228	***	0.150	0.141	0.253	***
TAM	CCL2	0.283	***	0.245	0.015	0.296	***
	CD68	0.185	***	− 0.061	0.552	0.244	***
	IL10	0.205	***	0.147	0.148	0.217	***
M1 Macrophage	INOS (NOS2)	0.230	***	0.092	0.365	0.299	***
	IRF5	0.126	*	− 0.219	0.030	0.215	***
	COX2(PTGS2)	0.079	0.073	0.303	*	0.037	0.444
M2 Macrophage	CD163	0.217	***	0.092	0.369	0.243	***
	VSIG4	0.230	***	0.057	0.577	0.264	***
	MS4A4A	0.231	***	0.119	0.243	0.253	***
Neutrophils	CD66b (CEACAM8)	0.054	0.217	0.187	0.065	0.051	0.297
	CD11b (ITGAM)	0.234	***	0.121	0.234	0.283	***
	CCR7	0.131	*	0.051	0.616	0.171	**
Natural killer cell	KIR2DL1	0.032	0.460	− 0.126	0.216	0.079	0.104
	KIR2DL3	− 0.050	0.259	− 0.213	0.035	− 0.003	0.945
	KIR2DL4	− 0.031	0.485	− 0.150	0.141	0.006	0.895
	KIR3DL1	− 0.079	0.073	− 0.244	0.016	− 0.021	0.667
	KIR3DL2	0.069	0.116	− 0.087	0.394	0.124	0.011
	KIR3DL3	− 0.042	0.340	− 0.029	0.775	− 0.032	0.519
	KIR2DS4	− 0.050	0.258	− 0.099	0.334	− 0.024	0.622
Dendritic cell	HLA-DPB1	0.115	*	− 0.033	0.744	0.158	*
	HLA-DQB1	0.092	0.035	− 0.046	0.652	0.128	*
	HLA-DRA	0.133	*	− 0.040	0.698	0.186	**
	HLA-DPA1	0.141	*	− 0.022	0.831	0.186	**
	BDCA-1(CD1C)	0.137	*	0.077	0.453	0.163	*
	BDCA-4(NRP1)	0.283	***	0.198	0.050	0.289	***
	CD11c (ITGAX)	0.224	***	0.075	0.462	0.263	***
Th1	T-bet (TBX21)	0.034	0.444	− 0.119	0.242	0.089	0.068
	STAT4	0.265	***	0.103	0.311	0.303	***
	STAT1	0.043	0.323	0.053	0.606	0.036	0.457
	IFN-γ (IFNG)	− 0.057	0.190	− 0.155	0.128	− 0.019	0.700
	TNF-α (TNF)	0.159	**	0.255	0.011	0.145	*
Th2	GATA3	0.023	0.598	0.057	0.578	0.022	0.653
	STAT6	0.128	*	0.070	0.492	0.156	*
	STAT5A	0.160	**	− 0.132	0.195	0.260	***
	IL13	0.029	0.514	0.023	0.826	0.044	0.365
Tfh	BCL6	0.275	***	0.026	0.799	0.347	***
	IL21	0.028	0.519	-0.035	0.734	0.061	0.209
Th17	STAT3	0.223	***	0.240	0.017	0.251	***
	IL17A	0.075	0.086	0.140	0.169	0.087	0.074
Treg	FOXP3	0.241	***	0.241	0.017	0.273	***
	CCR8	0.295	***	0.332	**	0.307	***
	STAT5B	0.271	***	0.056	0.582	0.320	***
	TGFβ(TGFB1)	0.216	***	0.191	0.060	0.218	***
T cell exhaustion	PD-1 (PDCD1)	0.023	0.600	− 0.111	0.275	0.077	0.114
	CTLA4	0.131	*	0.079	0.442	0.158	*
	LAG3	− 0.006	0.895	− 0.055	0.590	0.021	0.664
	TIM-3 (HAVCR2)	0.201	***	0.070	0.493	0.240	***
	GZMB	− 0.045	0.303	− 0.114	0.261	− 0.015	0.754

r: Value of Spearman’s correlation (*P < 0.05, **P < 0.001, ***P < 0.0001)

Interestingly, our results also suggested a close relationship between LAYN expression and immune cell markers of M2 macrophage in HPV-negative HNSCC (P < 0.001). Moreover, high expression of LAYN was also associated with CTLA4 and TIM-3 (P < 0.05) (Table 2), which could act as crucial genes in regulating T cell exhaustion. These results further confirmed that LAYN played a vital role in immune escape and immunomodulation.

### GO and KEGG enrichment analysis of LAYN target genes

**Fig. 4:**
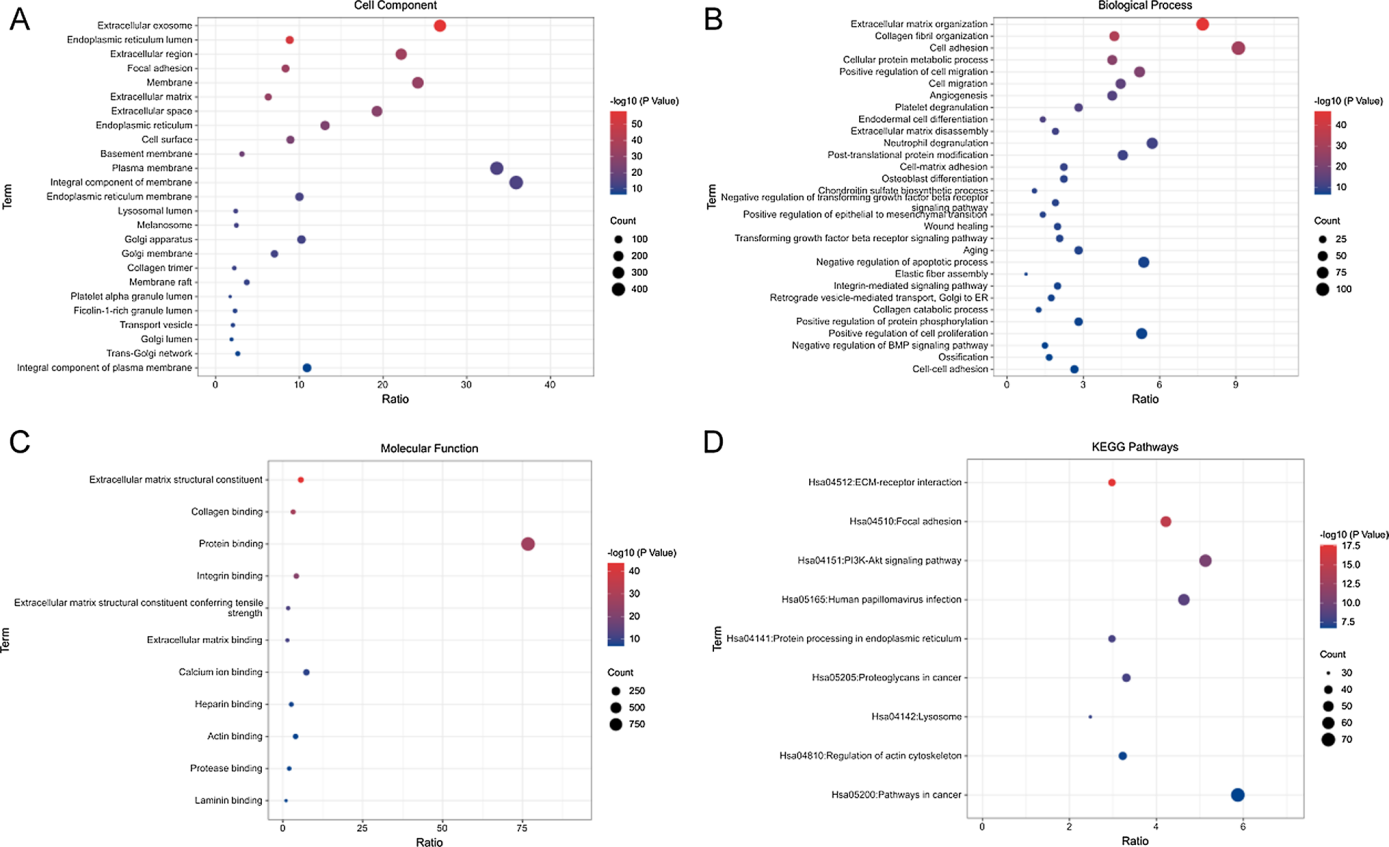
**A**–**C** GO and **D** KEGG Enrichment analysis of LAYN-related pathways

We performed GO and KEGG analyses to identify LAYN-related pathways. As was shown in Fig. 4A, LAYN-related gene groups were predominantly enriched in exosomes, extracellular regions, cell membranes, plasma membranes, and membrane components based on analysis of cellular component (GO-CC). In terms of Biological Process (GO-BP), the most enriched processes were extracellular matrix reconstruction, collagen fiber reconstruction, cell adhesion, cell protein metabolism process, and positive regulation of cell migration (Fig. 4B). The most enriched terms for molecular function (GO-MF) were extracellular matrix construction, collagen binding, and protein binding (Fig. 4C). KEGG pathway analysis suggested that LAYN co-expression network was mainly enriched in ECM receptor interaction, adhesive plaque, PI3K-Akt signaling pathway, HPV infection, and cancer-related pathways (Fig. 4D).

### The expression and prognosis patterns of HPV and LAYN in HNSCC patients

We evaluated the expression of HPV and LAYN in a total of 61 HNSCC tissues through SP immunohistochemistry. Baseline characteristics of HNSCC patients are presented in Table 3. The judgment standard for histochemical results was based on the presence of brown-yellow granules in the cell membrane/cytoplasm of HNSCC cells. Five high-power fields of vision (× 400) were randomly selected, and 100 cells were counted per field to determine the percentage of positive cells (PP):  < 5% = 0 points, 5–25% = 1 point, 26–50% = 2 points, 51–75% = 3 points, and > 75% = 4 points. Staining intensity (SI) was scored as follows: no staining = 0 points, light yellow = 1 point, dark yellow = 2 points, and brown = 3 points. The final score was determined by multiplying the number of positive cells and the intensity of cell staining: total score of 0, 1–4 points, 5–8 points, and 9–12 points corresponded to negative, + , +  + , and +  +  + , respectively.

**Table 3:** Clinical and pathological features of HNSCC patients in the study

Variables	Number of cases (%)		
	Oral Cavity Cancer (44)	Oropharyngeal Cancer (11)	Other types (6)
Age (years)			
Median	65	68	58
Range	44–73	48–76	43–78
Gender			
Male	33 (75.0)	7 (63.64)	5 (83.33)
Female	11 (25.0)	4 (36.36)	1 (16.67)
TNM stage			
I–II	17 (38.64)	3 (27.27)	1 (16.67)
III–IV	27 (61.36)	8 (72.73)	5 (83.33)
Pathology			
Well	5 (11.36)	3 (27.27)	2 (33.33)
Moderate and poor	39 (88.64)	8 (72.73)	4 (66.67)
Metastasis location			
Liver	7 (15.9)	7 (63.64)	2 (33.33)
Lung	11 (25.0)	3 (27.27)	2 (33.33)
Lymph node	24 (54.55)	8 (72.73)	4 (66.67)

**Fig. 5:**
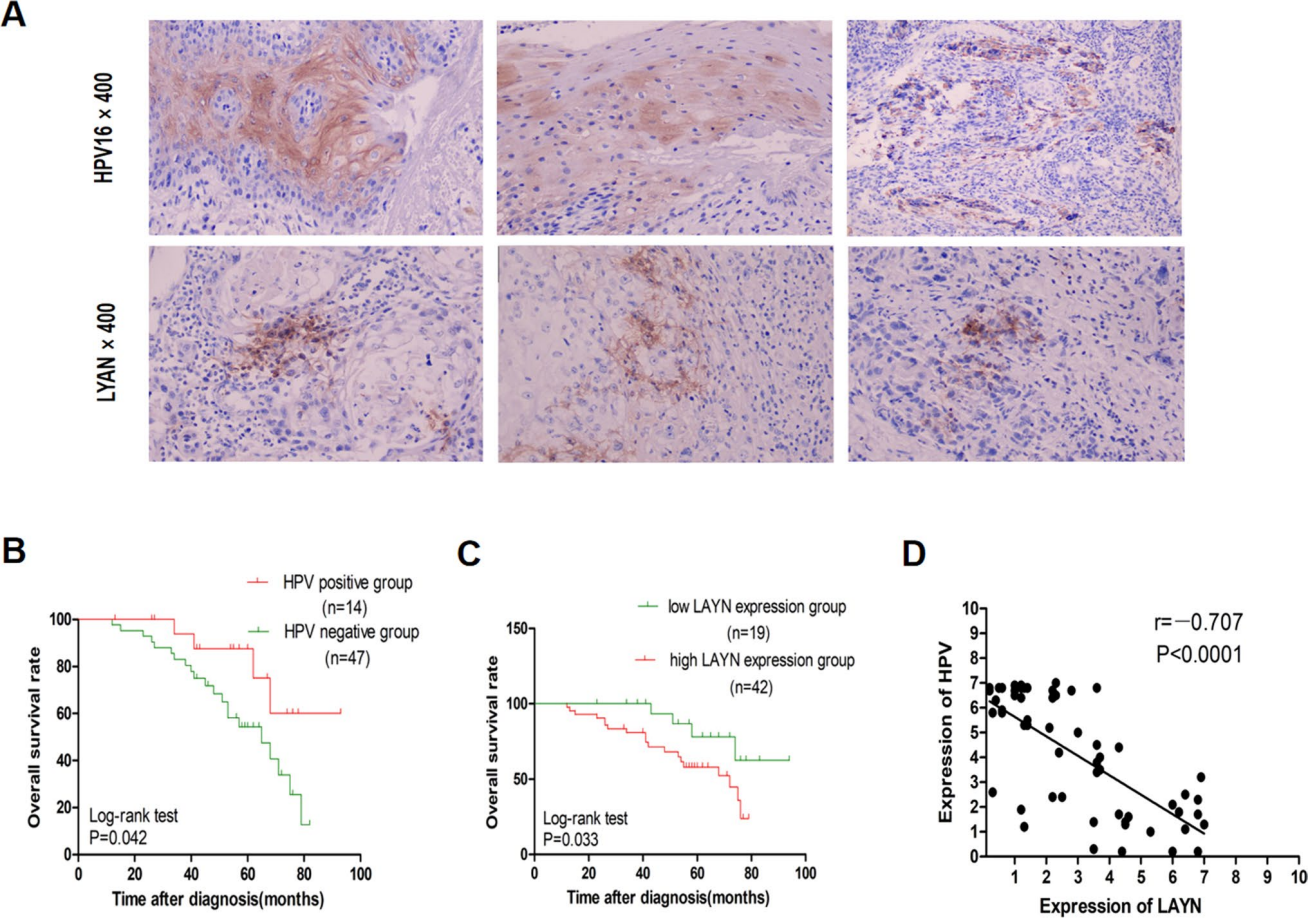
**A** Immunohistochemistry of the expression levels of HPV and LAYN in HNSCC patients. **B** OS survival curves of HPV-positive and HPV-negative HNSCC patients. **C** OS survival curves of LAYN-positive and LAYN-negative HNSCC patients. **D** Correlation analysis between HPV and LAYN expression levels

In 61 cases of HNSCC, 14 cases (22.95%) were HPV-positive, and 42 cases (68.85%) were LAYN-positive. All slides were reviewed by two investigators who recorded the percentage of cells positive for each stain (0 to + 4) and the intensity of stain (0 to + 3) (Fig. 5A).

In addition, we explored whether the expression levels of LAYN and HPV were correlated with the survival of patients with HNSCC. Using Kaplan–Meier methods, we found that HPV-positive group was correlated with prolonged OS of HNSCC patients (HR = 2.364, 95% CI 1.031–5.420, P = 0.042). In the meantime, a favorable prognosis was observed in reduced LAYN expression for HNSCC patients, which was in accordance with our previous results (HR = 2.508, 95% CI 1.075–5.851, P = 0.033) (Fig. 5B, C). Correlation analysis showed that the expression level of HPV was significantly negatively related to the expression level of LAYN (r = − 0.707, p < 0.0001).

## Discussion

LAYN is known to be one of the receptors for HA and involved in the reorganization of cytoskeleton structures [20]. The LAYN protein plays an important role in cell adhesion, migration, and cooperates with the body’s anti-tumor immunity [21]. Previous studies have shown that LAYN was correlated with poor prognosis and tumor immune cell infiltration in colorectal cancer [20], gastric cancer [14], and hepatocellular carcinoma [21]. However, up to now, no studies have reported the expression and prognostic value of LAYN in HNSCC, and the association of LAYN with immune infiltration in HPV-positive and HPV-negative HNSCC. In this study, we comprehensively analyzed the differential expression of LAYN in a diverse of tumors, the correlation of LAYN and HPV with prognosis, and the impact of LAYN on the immune microenvironment under different HPV statuses of HNSCC. Our study suggested that LAYN could serve as a significant biomarker to influence immune escape with a positive correlation with immune checkpoint molecules and TAMs, and lead to a poor prognosis in HPV-negative HNSCC.

We first explored the discordant expression of LAYN in pan-cancer tissues compared to their respective paracancerous tissues based on TCGA dataset, the results suggested the different tumor-specific roles of LAYN in different tumor types. In HNSCC, the expression of LAYN was significantly higher compared to adjacent normal tissues. Survival analysis showed that LAYN had a detrimental effect on overall survival of patients, which implied that LAYN might be a predictive factor of poor prognosis in HNSCC, especially in patients with the base of tongue cancer. Correlation analysis of clinicopathological factors indicated that LAYN expression was only related to HPV expression level. LAYN expression in HPV-positive HNSCC patients was significantly lower than that in HPV-negative HNSCC patients, and further analysis showed that HPV-positive HNSCC patients typically had better clinical outcomes than HPV-negative HNSCC patients, which was in consistent with our clinical sample results.

Tumor microenvironment (TME) is a complex and dynamic system consisting of various cellular components, including fibroblasts, stromal cells, endothelial cells, and immune cells [22, 23]. Immune cells from both the adaptive and innate immune systems infiltrate the TME, contributing to the modulation of tumor progression [24, 25]. Immune infiltration in the TME was reported to be an independent predictor of survival [26, 27]. To understand the potential mechanisms by which LAYN affects prognosis in HPV-related HNSCC, the relationship between LAYN expression and immune infiltration levels was investigated, and a direct correlation between LAYN and tumor-infiltrating immune cells was found in HNSCC, especially in HPV-negative HNSCC.

During further exploration of the role of LAYN in tumor immunity and its potential as an immunotherapeutic target, we analyzed the correlation between LAYN and 57 common immune marker sets. The results indicated a significant correlation between LAYN and 54.39% (31/57) of the immune marker sets in HPV-negative HNSCC. More specifically, LAYN expression was significantly correlated with markers of monocytes, TAMs, M1 macrophages, M2 macrophages, neutrophils, dendritic cells, and exhausted T cells in HPV-negative HNSCC. For instance, LAYN showed a positive correlation with genes CCL2, CD68, IL10, INOS, CD163, STAT3, and TGFβ with p values of less than 0.001.

T Cell Exhaustion refers to the loss of robust immune response functions in T cells, which is commonly observed in patients with chronic infections and cancer [28]. Prolonged exposure to persistent antigens or chronic inflammation causes exhausted T cells to gradually lose their effector function, and the characteristics of memory T cells also begin to diminish [29]. Our study has revealed a positive correlation between elevated expression of LAYN and CTLA4 as well as TIM-3, which were both immune checkpoint targets of exhausted T cells. Previous studies have reported that this exhaustion is reversible, at least partially, primarily through the blockade of inhibitory pathways such as PD-1 [30]. Immune checkpoint inhibitors effectively disrupt these signals, leading to a favorable anti-tumor effect by reversing T cell exhaustion and restoring the functionality of tumor infiltrating T cells within TME [31]. In clinical settings, the use of immune checkpoint inhibitors targeting exhaustion markers on T cells, such as PD-1, PD-L1 or CTLA-4, has demonstrated promising results [32–34]. Our findings provide a possible foundation for the development of therapeutic strategies for HPV-related HNSCC.

Interestingly, our results also revealed a strong relationship between LAYN expression and TAM/M2 macrophage infiltration in HPV-negative HNSCC. Macrophages can be classified into two subtypes, M1 macrophages and M2 macrophages, based on their functions and levels of inflammatory cytokine secretion [35]. M1 macrophages (classically activated macrophages) are primarily activated by lipopolysaccharide (LPS) and interferon-gamma (IFN-γ). They secrete high levels of IL-2 and relatively low levels of IL-10, playing a role in promoting inflammation, microbial killing, and phagocytosis [36]. On the other hand, M2 macrophages (alternatively activated macrophages) are mainly activated by the inflammatory cytokine IL-4 [37]. They suppress M1 macrophages by secreting anti-inflammatory cytokines like IL-10, and they play a role in wound healing and tissue repair processes [38]. Tumor-associated macrophages (TAMs) closely resemble M2-polarized cells, promoting an immune-suppressive microenvironment and ultimately leading to tumor growth, invasion, and metastasis [39, 40]. The imbalance of M1/M2 ratio plays a key role in the development of tumor immune escape, subsequent metastasis, therapy resistance and poor prognosis [41]. Further studies need to be conducted to determine whether LAYN is a crucial factor that mediates the M2 polarization of macrophages and ultimately results in tumor metastasis.

There were several limitations in this study. Firstly, more in vivo and in vitro experiments should be done to validate our findings. Secondly, the inconsistency of cut-off values among different online databases might introduce potential heterogeneity in the analysis.

To the best of our knowledge, this is the first study to report LAYN as a novel biomarker for HNSCC. Additionally, it sheds light on the association between the expression of LAYN and HPV, as well as the impact of LAYN on immune infiltration levels.

In conclusion, our findings suggested that LAYN played a crucial role in influencing the prognosis of HNSCC patients, likely through its interaction with infiltrating immune cells. With further understanding of its functional scope, the involved mechanism of LAYN might provide a novel prognostic biomarker and latent therapeutic target for the treatment of HPV-related HNSCC.

## Data Availability

The datasets used and/or analyzed during the current study are available from the corresponding author on reasonable request.

## Abbreviations

LAYN: Layilin
HNSCC: Head and neck squamous cell carcinoma
HPV: Human papillomavirus
TCGA: The Cancer Genome Atlas
TIMER: Tumor immune estimation resource
GEPIA: Gene expression profiling interactive analysis
OS: Overall survival
TAMs: Tumor-associated macrophages
HR: Hazard ratio
Tregs: Regulatory T cells
GTEx: Genotype-Twassue Expression
GO: Gene Ontology
KEGG: Kyoto Encyclopedia of Genes and Genomes
TME: Tumor microenvironment
DFS: Disease-free survival
